# Ecology of *Elodea canadensis* Michx. and *Elodea nuttallii* (Planch.) H. St. John—Insights from National Water Monitoring in Croatia

**DOI:** 10.3390/plants13121624

**Published:** 2024-06-12

**Authors:** Marija Bučar, Anja Rimac, Vedran Šegota, Nina Vuković, Antun Alegro

**Affiliations:** Division of Botany, Department of Biology, Faculty of Science, University of Zagreb, Marulićev trg 20/II, 10000 Zagreb, Croatia; marija.bucar@biol.pmf.hr (M.B.); vedran.segota@biol.pmf.hr (V.Š.); nina.vukovic@biol.pmf.hr (N.V.); antun.alegro@biol.pmf.hr (A.A.)

**Keywords:** macrophytes, Southeastern Europe, freshwater habitat, invasive plants, water monitoring

## Abstract

*Elodea canadensis* Michx. (common waterweed) and *Elodea nuttallii* (Planch.) H. St. John (Nuttall’s waterweed), two invasive aquatic plants from North America, have coexisted in European water bodies since the early 20th century. New localities for both species in Croatia continued to be discovered during a study that ran from 2016 to 2023 as a part of the annual implementation of Water Framework Directive monitoring that covered the entire territory of Croatia (786 sampling points in total). Based on these data, the distribution and ecology of both species were analysed. *Elodea canadensis* was found at 30 sampling points, mostly in rivers, and *E. nuttallii* at 15 sampling points, mostly in artificial canals. Nearly three-quarters (72.5%) of all elodea sampling points were in the Pannonian Ecoregion. *Elodea canadensis* was discovered for the first time in the Continental—Dinaric and Mediterranean—Dinaric Subecoregions. To study the ecology of the species, for each sampling point, vegetation relevés were performed and monthly measurements of physico-chemical parameters were collected. The most common accompanying species for both elodeas are presented, and the difference in species assemblages between the sites with *E. canadensis* and *E. nuttallii* was confirmed with the ANOSIM test. Furthermore, Indicator Species Analysis revealed eight species characteristic of *E. canadensis* sites and eleven species characteristic of *E. nuttallii* sites. Fitting multivariate models (CCA and NPMR) to species abundance revealed the ecological reaction of *E. canadensis* and *E. nuttallii* to environmental descriptors. The most strongly contributing environmental descriptors that influence the distribution of both *Elodea* species are biochemical oxygen demand, electrical conductivity and total phosphorus. In Croatia, the replacement of *E. canadensis* with *E. nuttallii* was observed in several water bodies with high nutrient loads.

## 1. Introduction

In the light of the ongoing spread of alien invasive species, more and more research is focused on revealing the ecology of such plants. Invasive alien aquatic species have numerous traits, such as allelopathy, phenotypic plasticity and propagule pressure, that make them more competitive than native species and can be considered their mechanisms of invasion [1]. When successfully occupying a habitat, invasive plants become a threat to the stability of ecosystems by causing a loss of biodiversity, modifying the trophic structure and even changing overall habitat properties. When quantified, the management and damage costs of invasive alien species in Europe in the last 60 years add up to over a hundred billion euros [2]. In comparison to terrestrial plants, an increased proportion of aquatic plants have ecological and economic impacts on their habitats [3]. In Europe, the most widespread alien aquatic plant family is *Hydrocharitaceae*, with *Elodea canadensis* being the most widespread and *E. nuttallii* being the fourth most widespread species [4]. Hence, waterweeds are some of the most researched aquatic freshwater invasive plants in Europe [5,6,7,8,9,10,11,12,13,14,15].

The genus *Elodea* consists of nine accepted species of submerged, freshwater perennials spread throughout the world, with records from all continents except Antarctica. All species are native to either North or South America. Out of the three species that have been introduced to Europe, *E. canadensis* and *E. nuttallii* are both native to temperate North America [16] and express invasive character all over the continent [17]. One of the main drivers of the spread of waterweeds in the introduced areas is their vegetative reproduction in the absence of male plants. After fragmentation, the detached stem parts quickly develop adventitious roots and continue the expansion of the species. Both species have also been recorded in Croatia—*E. canadensis* was first recorded near the city of Sisak by Josip Schlosser, presumably before 1883, as the collector died in 1882 [18]; *E. nuttallii* was first recorded in 2006, in the drainage canals of Kopački Rit [9].

To date, research on waterweeds in Croatia has been very limited. Košćec [19] reported a record of *E. canadensis* in the surroundings of the city of Varaždin in 1909, the first published record of the species (Northern Croatia). He was, however, probably unaware of the two unpublished herbarium specimens stored in the Herbarium Croaticum—ZA collection, previously collected by Josip Schlosser around Sisak (Central Croatia) (ZA152740) and by Stjepan Gjurašin, who recorded the species in Ješkovo Pond (Northern Croatia) (ZA152740) [20]. In his paper, Košćec [19] gave a detailed overview of the habitat and accompanying species. He assumed that *E. canadensis* was introduced into Croatian waterways from the northwest via the Drava River, since it was found in one of its tributaries. After this paper, *E. canadensis* was sporadically reported from the waterways and ponds of the Pannonian Ecoregion [21] for over a century, after which a new, *Elodea*-centred research paper was published by Kočić et al. [9] following the first discovery of *E. nuttallii* in Croatia in 2006. The distribution and morphological variations of both waterweeds were presented by the authors, and the further spread of *E. nuttallii* in Croatia was anticipated. In 2018, the first record of the previously congeneric *Egeria densa* Planch (syn. *Elodea densa* (Planch.) Casp.) in Croatia was published by Rimac et al. [22] updated by Vuković et al. [23], who presented the results of extensive mapping, showing a large number of localities spread across a much wider area than first reported. Finally, Piria et al. [24] classified *E. nuttallii* as very high risk and *E. canadensis* as high risk in terms of their invasiveness under current climate conditions in the Pannonian and Mediterranean regions of Croatia, as determined using risk-screening tools.

This paper gives the latest overview of the distribution of both waterweeds in Croatia and the first analyses of their ecology in the area. Resulting from the ecological studies that include the species samples from the whole of the country, this research contributes to the overall knowledge of the species’ environmental preferences.

The aims of this research were to do the following: (a) present the latest observations of *E. canadensis* and *E. nuttallii* in Croatia; (b) analyse both species’ preferences in terms of habitat characteristics; (c) analyse both species’ response to water chemical and physico-chemical parameters; and (d) present the most common accompanying species.

## 2. Results

### 2.1. Distribution

**Figure 1:**
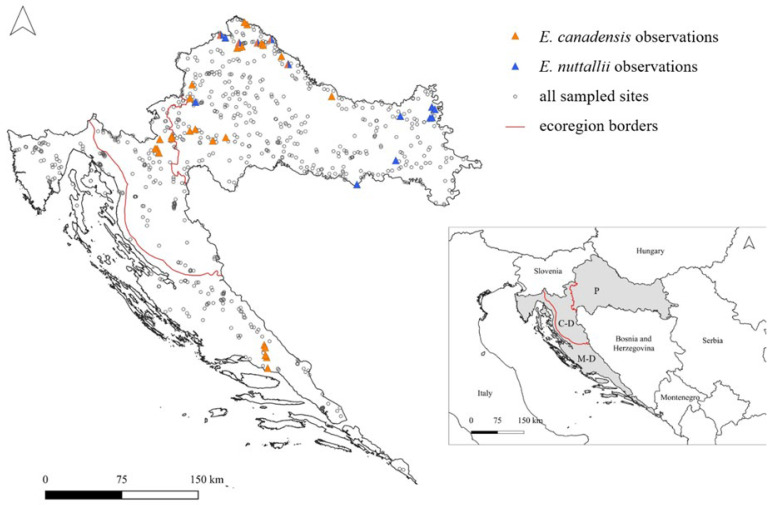
Distribution of *Elodea canadensis* and *E. nuttallii* in Croatia; lower right map shows the position of Croatia (grey) in Southeastern Europe (P—Pannonian Ecoregion, C-D—Continental–Dinaric Subecoregion, M-D—Mediterranean–Dinaric Subecoregion).

During the period 2016–2023, waterweeds were recorded at 40 sites (Figure 1) out of the 786 sampled—24 rivers, 12 canals and 4 reservoirs. *Elodea canadensis* and *E. nuttallii* were exclusively recorded at 25 and 10 sampling sites, respectively. Furthermore, at 5 sampling sites, they were both recorded, but in different years, never accompanying one another. Over the years, *E. nuttallii* replaced *E. canadensis* in three reservoirs on the Drava River in Northern Croatia (sites number 33–38) and in two canals of the Drava River Basin (sites number 31, 32, 43, 44). Overall, waterweeds were mostly recorded in rivers and canals (90% of records). *Elodea canadensis* was recorded in 22 rivers, 4 canals and 4 reservoirs, while *E. nuttallii* was recorded in 2 rivers, 10 canals and 3 reservoirs. Most records are in the Pannonian Ecoregion (72.5% of records), followed by the Continental–Dinaric Subecoregion (15.0%) and the Mediterranean–Dinaric Subecoregion (12.5%). During the study, *E. canadensis* was found for the first time in the Dinaric Ecoregion: first in the Continental Subecoregion in 2016, followed by a record in the Mediterranean Subecoregion in 2017. On the other hand, *E. nuttallii* was recorded for the first time in the Sava River Basin and is still present only in the Pannonian Ecoregion. Out of 40 sampling sites where waterweeds were recorded, only four are represented by a true lentic habitat in the form of a reservoir. 

### 2.2. Habitat Characteristics

Assessment of certain abiotic characters of the habitat (average water depth, turbidity, flow velocity, illumination/canopy coverage and substrate size) showed the preference of both waterweeds for water bodies with an average water depth of over 30 cm, clear to moderately turbid water, stagnant to moderately turbulent flow velocity and full illumination, with finer substrate (particles smaller than 2 cm) (Table 1). Furthermore, the difference between *E. canadensis* and *E. nuttallii* can be observed in their preferences with respect to flow velocity and substrate size. *Elodea canadensis* showed a preference for moderately turbulent watercourses (57% of records), whereas *E. nuttallii* was recorded more frequently in stagnant water (56% of records). Furthermore, *E. nuttallii* grew predominately in watercourses with clay and mud (81% of records), while *E. canadensis* grew on clay and mud (36%), sand and small pebbles (37%) and pebbles (17%). A slight difference in preference for average water depth can be seen as well—*E. nuttallii* was recorded evenly in water bodies with average depths of 30–100 cm and over 100 cm, while *E. canadensis* preferred water bodies with an average water depth of more than 100 cm in 71% of records. All of the characteristics were tested using the Mann–Whitney test. Here, substrate sizes were tested separately because, in each site, the substrate was evaluated as a fraction of each size category. The test showed that the sites with *E. canadensis* and *E. nuttallii* significantly differed in flow velocity (*p* = 0.023) and the content of the smallest substrate size (clay and mud) (*p* = 0.007).

### 2.3. Accompanying Species

**Figure 2:**
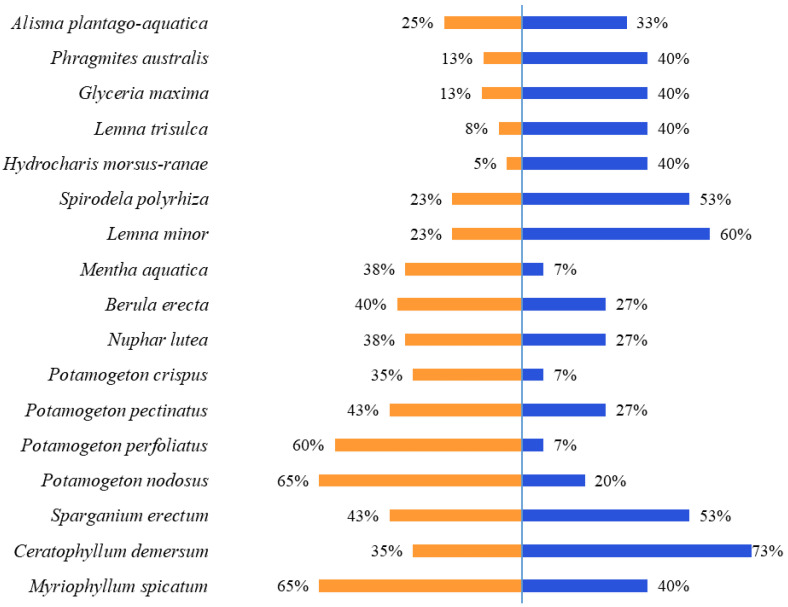
A list of the most frequent (>30% frequency) species accompanying *E. canadensis* (orange) and *E. nuttallii* (blue).

A total of 80 species were recorded alongside the two waterweeds during our study. Vegetation relevés are given in Table S1. *Elodea canadensis* was accompanied by 68 species and *E. nuttallii* by 46 species. Only three species frequently accompany both *E. canadensis* and *E. nuttallii* (in over 30% of relevés)—*Ceratophyllum demersum*, *Myriophyllum spicatum* and *Sparganium erectum*. *Elodea canadensis* is also frequently accompanied by *Potamogeton nodosus*, *P. perfoliatus*, *P. pectinatus*, *P. crispus*, *Berula erecta*, *Mentha aquatica* and *Nuphar lutea*. *Elodea nuttallii*, on the other hand, is most frequently accompanied by the free-floating *Lemna minor*, *L. trisulca*, *Hydrocharis morsus-ranae* and *Spirodela polyrhiza* alongside the helophytes *Glyceria maxima*, *Phragmites australis* and *Alisma plantago-aquatica* (Figure 2). Bryophytes were recorded in 19 relevés almost exclusively alongside *E. canadensis*, except for two relevés with only one bryophyte, *Riccia fluitans*, accompanying *E. nuttallii*. The most frequent bryophytes were *Fontinalis antipyretica* (24%) and *Cinclidotus fontinaloides* (13%), while the rest were recorded in less than 9% of relevés. Algae were represented by five stonewort species (Charales) in eight relevés, each with a frequency of up to 5%, and exclusively accompanying *E. canadensis* in our study (Table S1). 

An ANOSIM test showed that there was a significant difference between the sites with *E. canadensis* and *E. nuttallii* considering the composition and abundance of the species (ANOSIM, R = 0.345, *p* = 0.001, 999 permutations). Furthermore, Indicator Species Analysis identified a group of eight species characteristic of the sites with *E. canadensis* (*Potamogeton nodosus*, *P. perfoliatus*, *P. berchtoldii*, *P. crispus*, *Myriophyllum spicatum*, *Fontinalis antipyretica*, *Mentha aquatica* and *Ranunculus trichophyllus*) and eleven species indicative of *E. nuttallii* sites (*Ceratophyllum demersum*, *Lemna minor*, *L. minuta*, *L. trisulca*, *Spirodela polyrhiza*, *Hydrocharis morsus-ranae*, *Phragmites australis*, *Glyceria maxima*, *Nymphoides peltata*, *Salvinia natans* and *Typha latifolia*) (Table S2).

### 2.4. Water Chemical and Physico-Chemical Parameters

**Figure 3:**
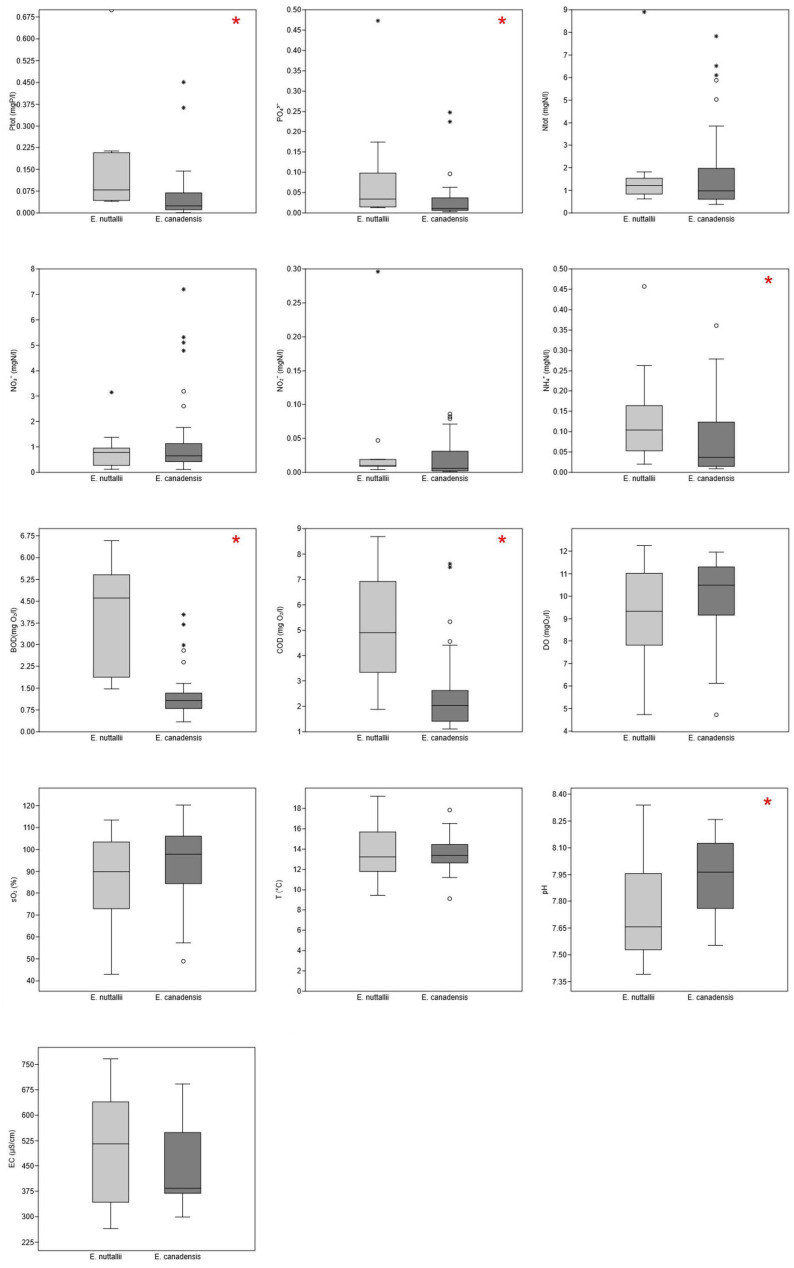
Boxplots of chemical and physico-chemical variables for sites with waterweeds. Total phosphorus—Ptot (mgP/L), orthophosphates—PO₄^3^^−^ (mgP/L), total nitrogen—Ntot (mgN/L), nitrates—NO₃^−^ (mgN/L), nitrites—NO₂^−^ (mgN/L), ammonium—NH₄⁺ (mgN/L), biochemical oxygen demand—BOD (mgO₂/L), chemical oxygen demand—COD (mgO₂/L), dissolved oxygen—DO (mgO₂/L), oxygen saturation—SO₂ (%), temperature—T (°C), conductivity—EC (μS/cm). A red asterisk in the upper right corner of a boxplot indicates a significant difference (*p* < 0.02) between variables (Mann–Whitney test) in sites with *E. canadensis* and *E. nuttallii*. (outliers: o—“out” values, *—“far out” or extreme values).

The results of the Mann–Whitney test showed a significant difference (*p* < 0.05) between sites of *Elodea nuttallii* and *E. canadensis* sites for six water parameters: total phosphorus, orthophosphates, ammonium, pH and chemical (COD) and biochemical (BOD) oxygen demands (Figure 3). The sites did not differ regarding total nitrogen (Ntot), nitrates (NO₃^−^), nitrites (NO₂^−^), dissolved oxygen (DO), oxygen saturation (sO₂), temperature (T) and conductivity (EC). *Elodea nuttallii* occurred in water with much higher BOD and COD, within ranges 1.5–6.6 mgO_2_/L and 1.9–8.7 mgO_2_/L respectively, whereas the median for sites of *E. canadensis* for BOD and COD lie approximately at the minimum of those values for *E. nuttallii* (1.07 mgO_2_/L for BOD and 2.03 for COD mgO_2_/L). Regarding pH, *E. nuttallii* showed a broader range but had a much lower median (7.66) in comparison with *E. canadensis*, whose median value of 7.96 exceeded the upper quartile of values for *E. nuttallii*. In terms of nutrient concentrations, sites of *E. canadensis* showed significantly lower values for ammonium, orthophosphate and, consequently, total phosphorus values. *Elodea nuttallii* was recorded in more eutrophic water, tolerating extreme values of total phosphorus up to 0.699 mgP/L and ammonium of 6.065 mgN/L (the latter value for ammonium was removed from the boxplot as an extreme outlier for visual purposes). All averages of environmental parameters of sample sites are given in Table S3.

### 2.5. Results of Ordination Analyses

**Figure 4:**
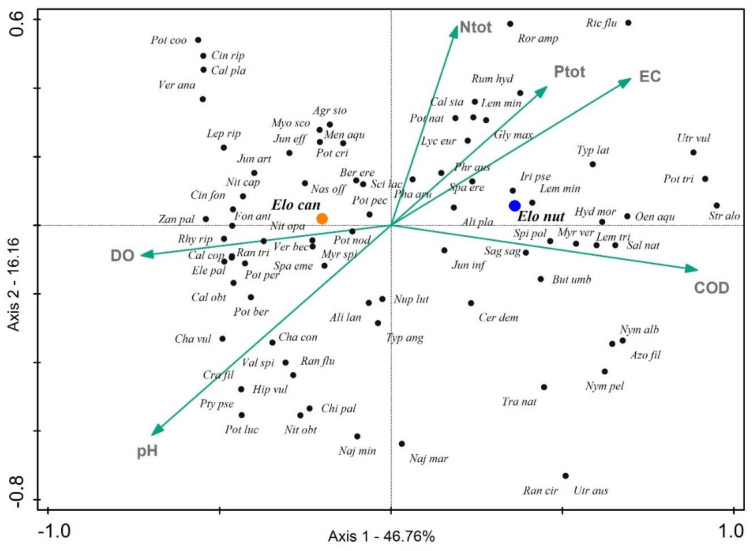
CCA ordination plot. The eigenvalues for the first and second axes equalled 0.41 and 0.14, respectively. Overall analysis was statistically significant (*p* < 0.002), which was confirmed by the Monte Carlo test (499 permutations). For environmental variables abbreviations, see the caption of Figure 3. For abbreviations of species’ names, see Table S1.

Results of the CCA (Figure 4) revealed the distribution pattern of waterweeds and accompanying species. Forward selection of the 13 measured environmental variables (physico-chemical and chemical parameters; listed in Table S4) revealed the six most contributing variables explaining the species distribution in different sample sites—total nitrogen, total phosphorus, electrical conductivity, chemical oxygen demand, dissolved oxygen and pH. The main compositional gradient, which can be seen along the first axis, clearly follows the increase in nutrient loads (total nitrogen, total phosphorus, electrical conductivity) and chemical oxygen demand, as well as a decrease in pH value. Hence, the less disturbed sites of lower trophy levels (high dissolved oxygen and pH values) on the left half of the plot are in contrast to the nutrient-rich, eutrophic sites on the right. This analysis clearly separates *E. canadensis* and *E. nuttallii* in different ecological niches, at least as observed in Croatian watercourses. *Elodea canadensis* shows an affinity for less eutrophic waters, while *E. nuttallii* grows in nutrient-rich watercourses with a high trophy level. Accompanying species of waterweeds are distributed accordingly. Furthermore, COD highly correlated with BOD (rs = 0.83, *p* < 0.001), while BOD also correlated with NH₄⁺ (rs = 0.72, *p* < 0.001), PO₄^3^^−^ (rs = 0.72, *p* < 0.001) and Ptot (rs = 0.79, *p* < 0.001). Dissolved oxygen was highly correlated with SO₂ (rs = 0.96, *p* < 0.001).

### 2.6. Nonparametric Multiplicative Regression Analysis

**Figure 5:**
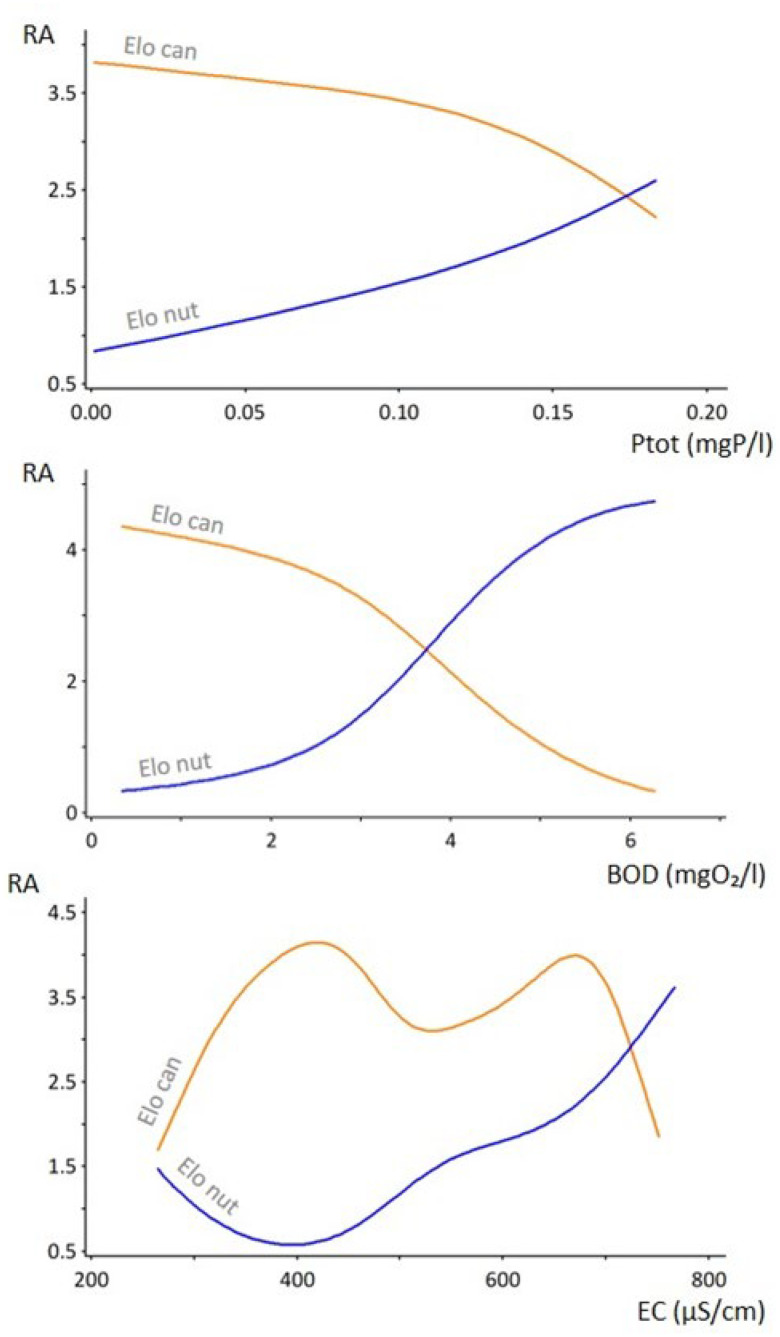
Non-parametric multiplicative regression. RA—relative abundance; Elo can—*Elodea canadensis*; Elo nut—*Elodea nuttallii*; Ptot (mgP/L)—total phosphorus; BOD (mgO₂/L)—biochemical oxygen demand; EC (μS/cm)—conductivity.

NPMR model identified the three most contributing predictors of the distribution of *E. canadensis* and *E. nuttallii*—biochemical oxygen demand, conductivity and total phosphorus (Figure 5, Table 2). Increasing values of the most relevant factor, total phosphorus, leads to a gradual increase in the relative abundance (RA) of *E. nuttallii* and a decrease in the RA of *E. canadensis*. *Elodea nuttallii* becomes relatively more abundant above a value of approximately 0.17 mgP/L of total phosphorus, which corresponds to hypereutrophic water bodies [25]. A similar relationship of the species’ RA can be seen with the increasing values of BOD. Interestingly, *E. canadensis* shows a bimodal response to conductivity, thriving in habitats with conductivity values of around 400 and 700 μS/cm but decreasing in abundance at 500–600 μS/cm and even more drastically after 700 μS/cm. The RA of *E. nuttallii*, on the other hand, increases gradually, with an increase in conductivity from 400 μS/cm onwards, and after 700 μS/cm it surpasses *E. canadensis* in abundance.

## 3. Discussion

*Elodea canadensis* was found for the first time in Croatia in the Pannonian Ecoregion (near the city of Sisak) in the late 19th century. The distribution of *E. canadensis* was further explored in the following century, but more sporadically than systematically [26,27,28,29,30]. It was not until the 2010s when *E. canadensis* was targeted as an invasive species that its distribution was more thoroughly investigated as a part of the inventory of invasive species [31] in Croatia. This information was deepened with the implementation of the WFD and the corresponding monitoring. *Elodea canadensis* was recorded across the Pannonian Ecoregion—in the basins of the rivers Sava, Drava and Danube. It was also found in the Continental-Dinaric Subecoregion for the first time in 2016, in the rivers Mrežnica and Dobra, both belonging to the Sava River Basin. The population of *E. canadensis* in these two sampling sites seems to be stable as has been confirmed for three and four consecutive years, respectively, in low abundances. Furthermore, in 2017, *E. canadensis* was also recorded in a canal connected to the Cetina River (Mediterranean Subecoregion) and subsequently in four locations along the river. Since there are not many tributaries, the species was presumably introduced in the Cetina either by human (disposal of aquarium contents) or bird activity.

A similar absence *of E. nuttallii* and isolated, scattered occurrences of *E. canadensis* in the Mediterranean biogeographical region of Italy was recorded by Buldrini et al. [32] who suggest that *E. nuttallii* is the less thermophilous of the two. A survey from neighbouring Serbia [33] found that most waterweed records have been documented in surface running waters as opposed to standing waters. This is also the case in our survey, even though in other parts of Europe, waterweeds are commonly recorded and studied in lakes [6,10,34,35]. The aforementioned new records of *E. nuttallii* in the Drava River Basin from 2019 are especially interesting since they match the monitoring sites where, only two years before, *E. canadensis* was observed. In these sites, *E. nuttallii* seemingly displaced *E. canadensis*, a possible scenario Kočić et al. [9] had predicted earlier for some water bodies. These sites were the reservoirs and two canals. Interestingly, *E. canadensis* is dispersed more widely in Europe and has been the more dominant species since it was introduced approximately a century before *E. nuttallii* [16], however, multiple research projects [11,14,35,36,37] showed that *E. nuttallii* spreads rapidly and consequently displaces *E. canadensis* in some areas. Assumed mechanisms of this displacement will be discussed further as these two species were first defined as ecologically and functionally redundant by having similar biological trait combinations and similar ecological responses, precisely the growth rate, to various environmental conditions and their combinations [38].

A study from Slovenia [39] found that *E. canadensis* was absent from the surveyed standing water bodies, whereas *E. nuttallii*, present in all water body types, dominated the ponds. *Elodea nuttallii* is poorly resistant to mechanical stress induced by water turbulence [40] and shows its invasive nature more in parts of the Drava River away from the main water flow [41]. Kuhar et al. [7] reported that *E. canadensis* is absent from water bodies with the most dynamic flow and prefers low-velocity currents in Slovenia and Thiébaut [13] reported similarly from north-eastern France. This is in accordance with our findings that *E. nuttallii* prefers stagnant flow when in running waters, and that neither of the two elodeas inhabit fast-flowing rivers. 

Both waterweeds grow in water bodies with more sediment of smaller size; however, *E. nuttallii* seems to prefer water bodies with finer sediment in contrast to *E. canadensis*, which is equally found in those with coarser substrates such as sand and gravel. Accordingly, Kuhar et al. [7] report the preference of *E. canadensis* for a mixture of gravel, sand and silt and Grudnik et al. [41] assume that the deposition of silt might contribute to the further expansion of *E. nuttallii* in reservoirs. Similarly, Crane et al. [35] report on a higher proportional cover of *E. nuttallii* on finer substrate, while Thiébaut [13] found *E. nuttallii* in habitats with high sand sedimentation. In Ukraine, in the central part of the Dnipro River Basin, *E. nuttallii* grows on sandy and muddy sediment, whereas *E. canadensis* grows on various substrates—sand, muddy sand, silt and rocky sediment [42]. In contrast, Bubíková et al. [43] recorded both elodeas mostly on coarse and sandy substrate as opposed to gravel in the surface waters of Slovakia.

Not many studies examined the relationship between water depth and *Elodea* occurrence/abundance. The reason why *E. canadensis* was found in waterbodies with deeper water might lie in the fact that it was more often found in rivers as opposed to canals which are usually shallower. Barrat-Segretain and Cellot [44] found that *E. nuttallii* tolerates droughts for longer periods than *E. canadensis*. This might result in *E. nuttallii* inhabiting shallower water bodies than *E. canadensis* as shallower water bodies (in the same ecoregions) are more prone to drawdowns during droughts. However, contrasting results come from Prokopuk and Zub [42] who recorded *E. canadensis* in somewhat shallower water (0.1–1.0 m) than *E. nuttallii* (0.2–1.5 m).

A survey in Slovakia identified a total of 58 macrophyte species accompanying waterweeds, while the most frequent taxa in elodea-dominated relevés were *Myriophyllum spicatum*, *Ceratophyllum demersum*, *Lemna minor*, *Potamogeton crispus* and *P. pectinatus* [43], which is quite similar to our results. İkinci [8] reports the following most common co-occurring taxa for *E. canadensis* in Turkey—*Ceratophyllum* sp., *Chara* sp., *Myriophyllum spicatum* and *Potamogeton natans*. Kuhar et al. [7] report *Potamogeton* spp. as the most frequent accompanying species of *E. canadensis* (*P. natans*, *P. nodosus*, *P. perfoliatus*, *P. crispus* and *P. pectinatus*). In the Greek part of Lake Prespa, *E. canadensis* was found accompanying *Myriophyllum spicatum*, *Trapa natans*, *Potamogeton perfoliatus*, *Ceratophyllum demersum*, etc. [45]. Such a list of accompanying species is in line with the high frequency of *E. canadensis* within the stands belonging to the vegetation of the class *Potamogetonetea*, as documented in Czechia [43]. Hence, it is not surprising that alongside *E. canadensis*, the most commonly recorded species in our survey were *Potamogeton* spp., and that *P. berchtoldii*, *P. crispus*, *P. nodosus*, *P. perfoliatus* were indicative of the sites with *E. canadensis* according to IAS analysis. On the other hand, the most common accompanying species of *E. nuttallii* were free-floating species, e.g., *Lemna* spp., *Spirodela polyrhiza*, *Hydrocharis morsus-ranae* inhabiting canals characterised by very slow flow, finer sediment and higher anthropogenic influence. These species were singled out as indicative of sites with *E. nuttallii*, alongside some less frequent species, such as *Salvinia natans* and *Nymphoides peltata*, and helophytes *Glyceria maxima*, *Phragmites australis* and *Typha latifolia*. It is widely known that free-floating plants belonging to vegetation of the class *Lemnetaea* often inhabit agricultural ditches and canals and are driven by nutrient enrichment, both nitrogen and phosphorus [46]. Our study confirmed that such habitats are also preferred by *E. nuttallii* in Croatia. Bryophytes and algae were not very frequent in the relevés, however, their distribution along the environmental gradient showed that they preferred sites with more oxygenated water, with higher pH and lower nutrient values, i.e., the sites with *E. canadensis* in our study. One of the bryophyte species, *Fontinalis antipyretica* was even shown to be indicative of sites with *E. Canadensis* within our study. The only species that accompanied *E. nuttallii* was *Riccia fluitans*. Rimac et al. [47] showed that among the water bryophytes in Croatia, only *R. fluitans* and *Leptodyctium riparium* had an affinity for hypereutrophic water with neutral pH, high electrical conductivity and organic matter content, although having wide niches considering these environmental parameters. Vanderpoorten et al. [48] also found that *L. riparium* exhibits a wide ecological range and is thus not a reliable indicator regarding trophy levels. Interestingly, in our study, *L. riparium* was not associated with hypereutrophic and eutrophic sites, but rather with oligo- and mesotrophic water of *E. canadesis* sites. Similarly, the stoneworts, known to have an affinity for oligotrophic conditions, which they help maintain by controlling nutrient cycles [49], were exclusively found on *E. canadensis* sites within our study.

Canonical correspondence analysis separated *E. nuttallii* and *E. canadensis* with their respective co-occurring species in terms of environmental niches. *Elodea canadensis* showed an affinity for more oxygenated water bodies with higher pH, as did its accompanying species. By contrast, *E. nuttallii* and its accompanying species showed a preference for water bodies with higher electrical conductivity, greater nutrient load (Ptot, Ntot) and higher chemical oxygen demand. The presence of *E. canadensis*, rather than of *E. nuttallii*, in sites with higher water pH might be explained by the fact that the photosynthesis rate of *E. nuttallii* reduces at pH > 7 [50]. This could also be the reason why *E. nuttallii* is not recorded in the oligo-mesotrophic and oxygenated waters of the Dinaric Ecoregion where the pH is more alkaline due to the karstic matrix. Even though both waterweeds show high growth rates and high tolerance to wide ranges of environmental variables, along with other ecological mechanisms of invasion (enemy release, allelopathy, phenotypic plasticity) [1] and are considered ecological equals, it has become more evident that *E. nuttallii* outcompetes *E. canadensis* in more eutrophic conditions, and the reasons behind that phenomenon are becoming more and more clear. Other than the previously mentioned higher tolerance to drawdowns [44], *E. nuttallii* has a higher relative growth in nitrogen-enriched water as shown by Ozimek et al. [34] and grows quicker in shadier and hypertrophic conditions, which allows it to outgrow and outcompete other species [12]. NPMR analysis supports the CCA by indicating that the most important environmental predictors of the distribution of waterweeds are the amount of total phosphorus, biochemical oxygen demand and water electrical conductivity, with high values indicating eutrophy. 

In our study, the displacement of *E. canadensis* by *E. nuttallii* was documented in five sampling sites, while the complete corresponding water chemistry was available only for two sites. These were characterized by high concentrations of orthophosphates and total phosphorus and ammonium, nitrates and total nitrogen (Table S3). These findings might suggest that *E. nuttallii* is more competitive in hypereutrophic situations and in general in those with a greater nutrient load. Furthermore, the possible displacement of *E. canadensis* by *E. nuttallii* was observed in our study on three reservoirs on the Drava River which might be due to the better tolerance of *E. nuttallii* to the drawdowns as well as the fast growth early in vegetation season underpinning its successful competition with other submerged species for light and nutrients. This was already suggested by Kunii [51], who found that *E. nuttallii* can grow even in the winter if the water temperature is above 4 °C. The invasion of *E. nuttallii* was documented earlier in reservoirs in the upper reach of the Drava in Slovenia, situated upstream from Croatia [41]. Here, the species developed large biomass in parts of the watercourse unexposed to the main current in warmer years with high winter and spring water temperatures. Furthermore, Wang et al. [52] found that *E. nuttallii* showed superior advantages in terms of growth, length and an increase in shoot number in late winter and spring over other alien species in China, such as *Egeria densa*. Both *Elodea* species are recorded alongside a number of native species, and they do not dominate the vegetation relevés in the watercourses. However, in the artificial impoundments, the contrary was noticed and these habitats could become major expansion hotspots [41]. Since *E. nuttallii* is listed as an invasive alien species of Union concern as of 2017 [53], future trends of its spread in Croatia should closely be monitored.

## 4. Materials and Methods

*Elodea canadensis* and *E. nuttallii* were recorded alongside other macrophytes within the national surface water monitoring scheme conducted from 2016 to 2023. Macrophyte vegetation is monitored to assess the ecological status of water bodies, as required by the Water Framework Directive (WFD) [54]. Sampling was performed at 786 sites according to the national methodology for macrophyte sampling [21]. Watercourses are surveyed along 100-metre-long transects from the banks or by zigzagging across the riverbed if possible. Standing water bodies are surveyed from a boat and also by walking along the banks. If unattainable by eye or hand, macrophytes are sampled with rakes at the end of a long pole or tied to a rope. Distribution maps were created using QGIS Desktop 3.4.9 software. Vegetation relevés are recorded following the extended Braun-Blanquet scale (r = one individual, + = up to 5 individuals, 1 = up to 50 individuals, 2 m = over 50 individuals, coverage < 5%, 2a = coverage 5–15%, 2b = coverage 15–25%, 3 =25–50%; 4 = coverage 50–75%; 5 = coverage over 75%) [55,56,57]. The nomenclature follows Euro + Med [58] for vascular plants, Hodgetts et al. [59] for bryophytes and AlgaeBase [60] for algae. 

According to the typology developed as a basis for the monitoring of surface waters [21], the territory of Croatia (56,594 km^2^) is divided into two hydrological and biogeographical regions—the Pannonian and the Dinaric Ecoregion, the latter being subdivided into Continental and Mediterranean Subecoregions. The Pannonian Ecoregion is situated in the continental part of Croatia between three large rivers—Sava, Drava and Danube, so its watercourses belong exclusively to the Black Sea Basin. It is composed of low-altitude alluvial and diluvial plains in between low, solitary mountain massifs. Geologically and lithologically, the Pannonian Ecoregion is characterised by silicate Quaternary deposits, while limestone is found only in the highest mountain areas. The climate is temperate, without a dry season, with warm summers in most of the territory (Cfb) and hot summers predominantly in the eastern part (Cfa). The Dinaric Ecoregion is characterised by the Dinarides, the largest uninterrupted karst system in Europe, so it is predominantly built of limestone and dolomite bedrock. Many of its rivers exhibit partly subterranean courses, and some of them in the Mediterranean–Dinaric Subecoregion also periodically dry out over the summer season. This subecoregion is characterized by temperate Mediterranean climate, with dry and hot summer months (Csa), while the Continental–Dinaric Subecoregion is characterised by a continental climate (Cfb) and constant river discharge levels [61,62]. Information on each sampling site (Table S5) also comprised water chemical and physico-chemical parameters of water, as well as habitat characters of the water body, listed in Table 3. Chemical and physico-chemical parameters were measured monthly by an accredited laboratory (Central Water Management Laboratory, Zagreb) and their annual average was used in the analyses. Dissolved oxygen, oxygen saturation, temperature, pH and electrical conductivity were measured in situ with a Hach HQ40D Portable Multi Meter (Hach, Loveland, CO, USA) under standard conditions, while chemical parameters were analysed in the laboratory from water samples. Habitat characters were assessed in situ only for running waters (rivers and artificial canals). Basic descriptive statistics of these habitat characters were calculated. The distribution of *E. canadensis* and *E. nuttallii* along the gradient of chemical and physico-chemical parameters was shown with box-plot graphs made using Past 4.16 software [63]. Furthermore, a significant difference between the parameters was tested for each environmental variable with the Mann–Whitney pairwise post hoc test. Non-parametric Mann–Whitney test was chosen because the majority of the variables did not have a normal distribution, previously tested with the Shapiro-Wilk test. Both tests were performed in Past 4.16 software.

The frequency of accompanying species was calculated for the sites with *E. canadensis* and those with *E. nuttallii* to explore the differences. Furthermore, a non-parametric ANOSIM test (Analysis of Similarities) was used to test the significant difference of sites with *E. canadensis* and *E. nuttallii* regarding the total species composition and their abundances. Indicators Species Analysis (ISA) was performed to identify species characteristic of each group of sites, i.e., the species group with *E. canadensis* and that with *E. nuttallii*. The statistical significances of the species indicator values were estimated by 9999 random reassignments (permutations) of sites across groups. Both analyses were performed in Past 4.16 software.

To explore the relationship between the environmental variables and patterns in the distribution of waterweeds and accompanying species, a direct ordination method, canonical correspondence analysis (CCA), was used. After removing the outliers, vegetation and environmental data from 50 localities were included in the analysis. CCA was selected because the response data were compositional with a 3.6 SD unit-long gradient [64]. A step-forward selection procedure in CANOCO 5 [64,65] was used to identify the most contributing and nonredundant subset of environmental predictors influencing the investigated species. Six variables with the highest conditional effect and with a 5% significance cut level (*p* < 0.05; Monte Carlo test, 499 permutations) were included. Before the analysis, rare species were down-weighted. 

Ecological niches of the species regarding the measured water parameters were explored using nonparametric multiplicative regression analysis (NPMR) performed in HyperNiche V2.3 [66]. To create multiple best models, a local mean with Gaussian weighting for both the response (species relative abundance) and the predictors (13 measured physico-chemical parameters) was used. A stepwise free search was employed to automatically find the best models with different combinations of environmental variables and by adopting default values for all remaining search criteria. For validation of the model, the predictive quality of the cross-R2 (×R2) was calculated based on the residual sum of squares (RSS), divided by the total sum of squares (TSS). The value of ×R2 ranges from 0 to 1, indicating no relation and perfect fit, respectively. The best model for each species was selected on account of the additional predictor (i.e., environmental) variable resulting in only a minimal increase (< 5%) of ×R2. The relative importance of a particular predictor within the model was evaluated using sensitivity analysis. The sensitivity of the model was estimated by calculating the tolerance as a proportion of variable range. Sensitivity values range from 0 to 1 and greater sensitivity indicates a higher influence of a particular variable in the model [67]. The significance of the selected models was tested using a Monte Carlo randomization test with 100 runs to calculate the probability value [67].

## 5. Conclusions

In Croatia, *E. canadensis* has historically been present much longer and is more widespread than *E. nuttallii*. It inhabits more sites in more water bodies in all three (sub)ecoregions. It shows an affinity for illuminated mesotrophic to eutrophic, moderately turbulent rivers, where it grows on different substrates in various depths. Most often, it grows with macrophytes such as *Potamogeton* spp., *Myriophyllum spicatum*, *Berula erecta*, etc., whereas *E. nuttallii* accompanies free-floating species such as *Lemna* spp., *Spirodela polyrhiza*, *Hydrocharis morsus-ranae* and submerged *Ceratophyllum demersum*. *E. nuttallii* is still poorly recorded in Croatia. It mostly inhabits the shallower, eutrophic, stagnant to slow-flowing canals and impoundments characterised by soft sediment in the Pannonian Ecoregion. 

The replacement of *E. canadensis* with *E. nuttallii* was observed in several water bodies with high nutrient loads, which may be related to the latter species greater tolerance of nutrient-rich and oxygen-depleted conditions. Future monitoring of these two species is expected to reveal further expansion of *E. nuttallii* in the eutrophic water bodies of the Pannonian Ecoregion of Croatia, especially in the case of further eutrophication.

## Figures and Tables

**Table 1:** An analysis of average water depth, turbidity, flow velocity, illumination and substrate size in water bodies where waterweeds were recorded. A significant difference between sites with *E. canadensis* and *E. nuttallii* is indicated with an asterisk (Mann–Whitney test, *p* = 0.023 for flow velocity and *p* = 0.007 for clay and mud).

		Both Species	*E. nuttallii*	*E. canadensis*
Average water depth	0–30 cm	17%	11%	21%
	30–100 cm	25%	44%	14%
	>100 cm	58%	44%	71%
Turbidity	clear	50%	44%	57%
	moderately turbid	42%	44%	43%
	turbid	8%	11%	7%
* Flow velocity	stagnant	30%	56%	14%
	slow-flowing	22%	22%	21%
	moderately turbulent	43%	22%	57%
	turbulent	4%		7%
Shading	fully illuminated (0–20% canopy coverage)	96%	100%	93%
	partially shaded (40–60% canopy coverage)	4%	0%	7%
Substrate size	* clay and mud (<0.063 mm)	54%	81%	36%
	sand and small pebbles (0.063–20 mm)	26%	8%	37%
	pebbles (2–6.3 cm)	10%	0%	17%
	stones (>6.3 cm)	5%	0%	8%
	artificial (concrete)	5%	11%	1%

**Table 2:** Summary of NPMR models for *Elodea* species to environmental predictors of highest explanatory power.

	*E. canadensis*		*E. nuttallii*	
×R	0.4978		0.6588	
N* (average neighbourhood size)	12.231		12.316	
*p* (randomization test)	<0.05		<0.05	
	Tolerance (%)	Sensitivity	Tolerance (%)	Sensitivity
Ptot	5.00	0.3572	5.00	0.4469
BOD	20.00	0.2837	15.00	0.3389
EC	25.00	0.2220	35.00	0.0538

**Table 3:** A list of measured chemical and physico-chemical parameters and assessed habitat characters with accompanying abbreviations and measurement units or categories.

CHEMICAL PARAMETERS			HABITAT CHARACTERS	
total nitrogen	Ntot (mgN/L)		Average water depth	0–30 cm
nitrates	NO₃^−^ (mgN/L)			30–100 cm
nitrites	NO₂^−^ (mgN/L)			>100 cm
ammonium	NH₄⁺ (mgN/L)		Turbidity	clear
total phosphorus	Ptot (mgP/L)			moderately turbid
orthophosphates	PO₄^3^^−^ (mgP/L)			turbid
PHYSICO-CHEMICAL PARAMETERS			Flow velocity	stagnant
pH				slow-flowing
temperature	T (°C)			moderately turbulent
conductivity	EC (μS/cm)			turbulent
dissolved oxygen	DO (mgO₂/L)		Shading	0–20% canopy coverage
oxygen saturation	sO₂ (%)			20–40% canopy coverage
biochemical oxygen demand	BOD (mgO_2_/L)			40–60% canopy coverage
chemical oxygen demand	COD (mgO_2_/L)			60–80% canopy coverage
				80–100% canopy coverage
			Substrate size	clay and mud (<0.063 mm)
				sand and small pebbles (0.063–20 mm)
				pebbles (2–6.3 cm)
				stones (>6.3 cm)
				artificial (concrete)

## Data Availability

The data presented in this study are available on request from the corresponding author.

## Supplementary Materials

The following supporting information can be downloaded at: https://www.mdpi.com/article/10.3390/plants13121624/s1, Table S1: Vegetation relevés; Table S2: Indicator Species Analysis results; Table S3: Chemical and physico-chemical variables measured in sample sites, Table S4: CCA Forward selection results; Table S5: Sample sites with accompanying information on the year of survey and coordinates.
